# Primary Vaginal Epithelioid Angiosarcoma: A Case Report and Literature Review of a Rare Neoplasm

**DOI:** 10.7759/cureus.39258

**Published:** 2023-05-20

**Authors:** Muhammad Ahmad, Laura Warren, Hnin Ingyin, Anam Naumaan, Annacarolina F Da Silva

**Affiliations:** 1 Pathology and Laboratory Medicine, Weill Cornell Medicine/New York-Presbyterian Hospital, New York, USA; 2 Pathology and Laboratory Medicine, Washington University School of Medicine, St. Louis, USA

**Keywords:** gynecologic pathology, gynecologic tract malignancy, radiation, pathology, epithelioid angiosarcoma

## Abstract

Angiosarcoma is a malignant neoplasm showing morphological or immunophenotypic evidence of endothelial differentiation with either a vascular or lymphatic origin. It has a strong predilection for skin and deep soft tissue. Angiosarcomas of the gynecologic tract are very uncommon, and very few cases have been described in medical literature up to this day. Primary vaginal angiosarcomas with no prior history of radiation are exceedingly rare. The epithelioid subtype of primary vaginal angiosarcomas is even more uncommon. Here we present a rare case of an epithelioid subtype of primary vaginal angiosarcoma in a 47-year-old woman with no prior history of radiation who presented with pelvic pain, malodorous vaginal discharge, and a vaginal mass.

## Introduction

Angiosarcomas account for about 1%-2% of all soft tissue sarcomas in adults [1]. The most common risk factors include long-term exposure to radiation or exogenous toxins, chronic lymphedema, and familial syndromes [2]. Although angiosarcomas most commonly arise from the skin, they can involve any anatomic location, including superficial or deep soft tissue and viscera [3]. Angiosarcomas are aggressive tumors with significant morbidity and a high mortality rate. They have a five-year survival rate of about 35% and a median survival of roughly seven months [4]. Angiosarcomas of the female genital tract are very uncommon. Most of these involve the ovaries (54%), followed by the uterus (34.4%), vagina (6%), and vulva (5%) [4]. Angiosarcomas arising in the vagina are particularly rare, and only 13 cases in total (including nine cases in English medical literature) have been reported so far. Most of these angiosarcomas arise in the setting of prior radiation for a gynecologic tract malignancy. Primary vaginal angiosarcomas with no history of radiation are exceedingly rare, and only six cases have been reported to date. The epithelioid variant of primary vaginal angiosarcoma can be a diagnostic pitfall because it doesn't exhibit the conventional spindle cell morphology and can be easily mistaken for a more common epithelial lesion of the vulva, such as a squamous cell carcinoma, because of its epithelioid appearance.

## Case presentation

**Figure 1:**
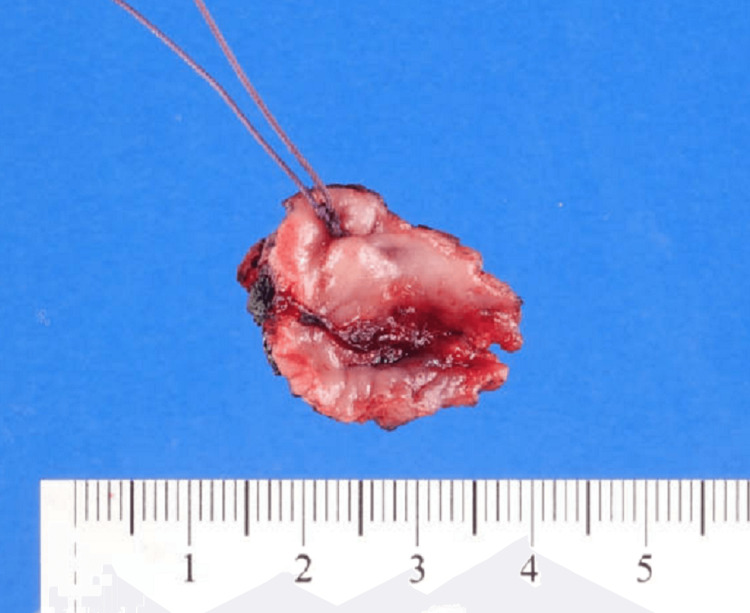
The partial vaginectomy specimen shows a 1.5 cm ulcerated lesion in the vaginal wall.

Our patient was a 47-year-old woman who presented with pelvic pain, dysmenorrhea, and malodorous vaginal discharge. On pelvic examination, a 1.4 cm pale, calcified, fungating, and necrotic-appearing lesion in the left vaginal wall was noted (Figure 1).

**Figure 2:**
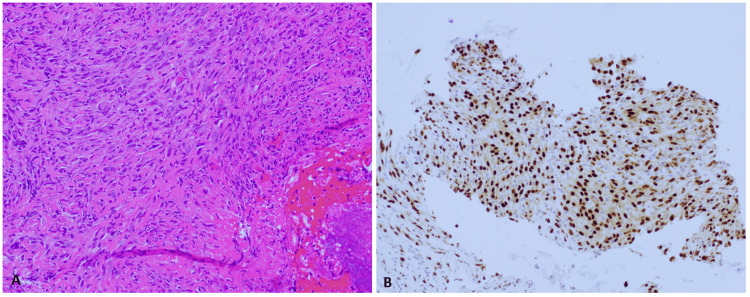
(2A) 200X; H&E, The biopsy specimen shows a neoplasm composed of spindled and epithelioid cells, focally forming cleft-like spaces and cytoplasmic vacuoles. (2B) 200X; ERG, The neoplastic cells show strong nuclear staining for ERG. H&E: hematoxylin and eosin stain

The lesion was biopsied, and the histologic examination showed a necrotic and ulcerated malignant neoplasm. The tumor showed spindled and epithelioid cells with atypical nuclei showing open chromatin and prominent nucleoli, focally forming cleft-like spaces and cytoplasmic vacuoles (Figure 2).

**Figure 3:**
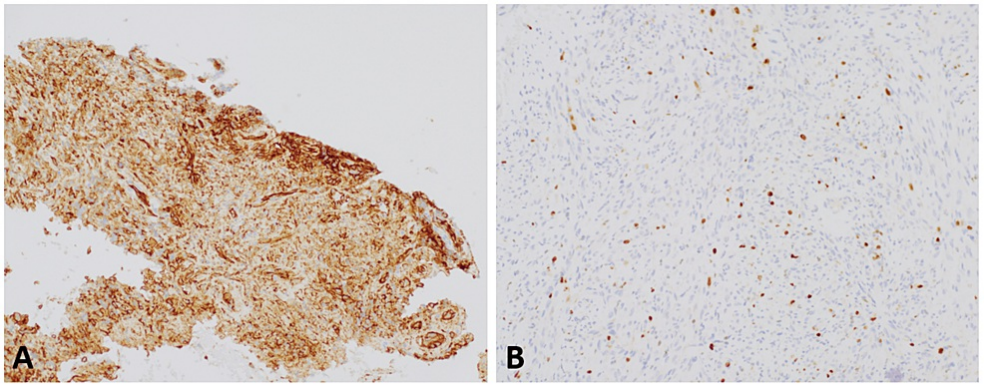
(3A) 200X; CD31, The neoplastic cells from the biopsy specimen show strong and diffuse membranous staining for CD31. (3B) 200X; Ki-67, The neoplastic cells from the biopsy specimen show a nuclear proliferation index of 5%-10%.

Immunohistochemical stains showed diffuse expression in the neoplastic cells for ERG and CD31 and focal positivity for c-myc, CAM5.2, and Ki-67 (5%-10% of tumor cells) (Figure 3).

The neoplastic cells showed no expression for ALK-1, CD34, HHV-8, desmin, SMA, p40, ER, PR, CAMTA1, and TFE3. Due to the extremely rare nature of the tumor and its epithelioid appearance, the case was also shared with soft tissue pathologists, and a diagnosis of angiosarcoma was favored. Therefore, complete excision of the lesion with wide margins was recommended.

**Figure 4:**
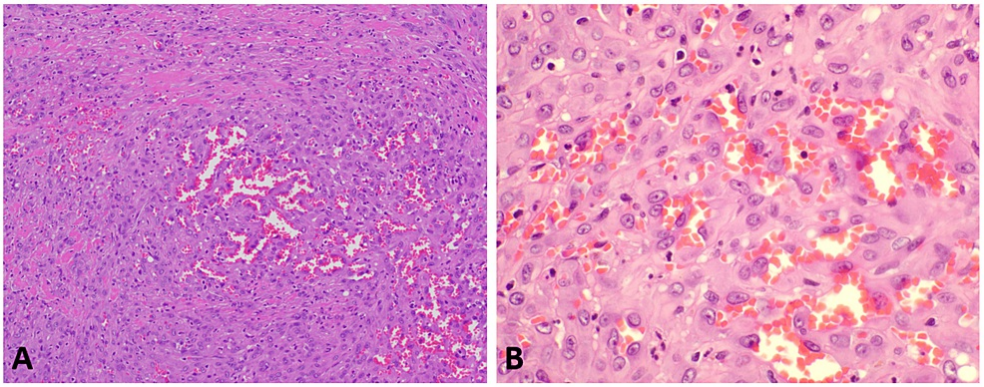
200X (4A) and 400X (4B); H&E, Neoplastic cells from the excision specimen show spindled and epithelioid morphology with atypical nuclei, open chromatin, and prominent nucleoli, forming cleft-like spaces with erythrocytes. H&E: hematoxylin and eosin stain

**Figure 5:**
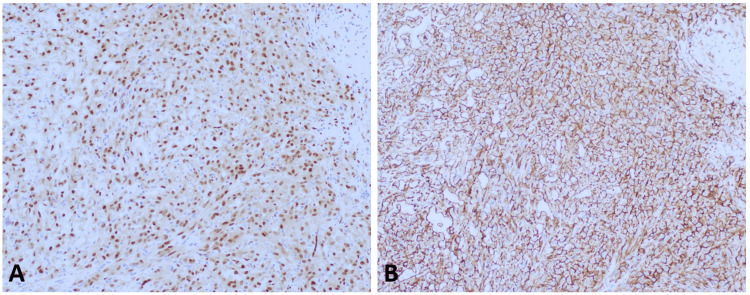
(5A) 200X; ERG: Neoplastic cells from the excision specimen show moderate and diffuse nuclear expression for ERG. (5B) 200X; CD31: Tumor cells from the excision specimen show strong and diffuse membranous expression for CD31.

The patient subsequently underwent a partial vaginectomy, which showed morphologic and immunophenotypic features similar to the biopsy (Figures 4-5).

Moreover, the neoplasm exhibited vasoformative areas, perineural invasion, foci of necrosis, and infiltrative growth at its periphery. The surgical resection margins were negative for tumors. Based on the clinical, morphologic, and immunophenotypic findings, a diagnosis of primary vaginal epithelioid angiosarcoma was confirmed. It has been almost five months since the patient's initial presentation, and she is currently undergoing radiation therapy.

## Discussion

Primary vaginal angiosarcomas with no prior history of radiation are exceedingly rare. An extensive literature review demonstrated that only six cases of primary vaginal angiosarcoma have been reported so far (Table 1). Prempree et al. were the first to describe a case of primary vaginal angiosarcoma in 1983 [5]. However, the neoplastic cells did not show any epithelioid features in that case. The first case of the epithelioid subtype of primary vaginal angiosarcomas was not reported until 1998 by McAdam et al. [6].

**Table 1:** Reported cases of primary vaginal angiosarcomas in the literature

Study (year)	Age	Clinical presentation	Epithelioid variant	Previous radiation for gynecologic tract malignancy	Previous hysterectomy	Follow-up and survival data
Prempree et al (1983) [5]	49	Vaginal mass	No	No	No	Disease-free at 36 months
McAdam et al (1998) [6]	86	Vaginal mass	Yes	No	Yes	four months
Kruse et al (2014) [4]	46	Vaginal mass, vaginal bleeding	No	No	No	48 months
Richer et al (2014) [7]	41	Vaginal mass	Yes	No	No	Disease-free at 30 months
Bratila et al (2016) [8]	22	Pelvic pain	Yes	No	No	Disease-free at nine months
Weishaupt et al (2021) [9]	66	Vaginal mass, vaginal bleeding	Yes	No	No	Six months
Current case (2023)	47	Vaginal mass, pelvic pain	Yes	No	No	Disease-free at five months

Based on the literature review, the age at diagnosis ranged from 22 years to 86 years (mean age: 51.66 years). Five of these six cases presented with a pelvic mass. Vaginal bleeding was the main presenting feature in two of the six cases, whereas pelvic pain was the main symptom in the case without a history of a vaginal mass [8]. A previous hysterectomy had been performed in only one of these six cases for a benign condition [6]. Four of these six cases showed epithelioid morphology in the neoplastic cells. Although conventional angiosarcomas may have focal epithelioid features, true epithelioid angiosarcoma is defined by a predominantly epithelioid morphology [8].

With only four previous cases reported in the literature, primary epithelioid vaginal angiosarcomas pose a significant diagnostic challenge because of their unusual location and non-specific clinical presentation. Angiosarcomas have a wide morphologic appearance, ranging from lesions that are cytologically bland and vasoformative to solid sheets of highly pleomorphic cells without definitive vasoformation. Based on morphology, the presence of subtle vascular features amidst a predominant epithelioid proliferation can be easily misdiagnosed and mistaken for an epithelioid lesion such as a squamous cell carcinoma. The cytological features to look out for in an epithelioid angiosarcoma include neoplastic cells with epithelioid morphology showing nuclear pleomorphism and mild-to-moderate cytologic atypia. Their architectural patterns can vary from nests and solid sheets to a trabecular or infiltrative growth pattern. The neoplastic cells usually have moderately eosinophilic cytoplasm with round nuclei and prominent nucleoli. Mitoses can range from very few to numerous. Because of the heterogeneous histologic features of poorly differentiated tumors, recognizing angiosarcomas can be extremely challenging. Their distinction from an epithelial neoplasm can be made by the presence of slit-like spaces, intracytoplasmic lumina containing red blood cells, and focal areas of irregularly anastomosing blood vessels.

In cases of diagnostic uncertainty, immunohistochemical stains are useful in differentiating epithelioid angiosarcomas from epithelial tumors. Epithelioid angiosarcomas of the vagina are usually positive for markers that are widely expressed in angiosarcomas (CD31 and CD34) and show a lack of expression for epithelial markers (cytokeratins), neural markers (S100), smooth muscle markers (myogenin, caldesmon, and desmin), mesothelial markers (calretinin and WT-1), and melanocytic markers (HMB-45 and Melan-A). These markers can aid the pathologist in distinguishing epithelioid angiosarcomas from epithelioid malignancies (which are in the differential diagnosis), such as carcinomas, malignant peripheral nerve sheath tumors, sarcomas, mesotheliomas, and melanomas. The underlying genetics of this particular entity are not fully established yet. Although MYC amplification is a frequent finding observed in secondary (radiation and lymphedema-associated) angiosarcomas, no specific genetic alterations have been reported in primary angiosarcomas thus far [8].

Once diagnosed, these tumors behave in a highly aggressive manner and have a very poor clinical course, with a five-year survival of 27%-35% and a median survival approximating seven months [4, 10]. Up to 50% of patients have metastases at the time of presentation [2]. Bones are the most common site of metastasis, followed by lymph nodes and the lungs. High rates of recurrence have also been associated with these tumors. Factors associated with the dismal prognosis for these tumors include advanced age, large size, increased mitotic activity, tumor necrosis, epithelioid morphology, and positive resection margins [11]. However, none of these associations have been validated by other studies.

There is a scarcity of evidence-based treatment recommendations available for vaginal angiosarcomas. The infrequency with which these tumors are observed contributes to the general lack of consensus on their optimal management. Due to the invasive and multifocal nature of angiosarcomas, the primary management of choice is radical surgical resection with wide and clear margins. A study of 51 patients with angiosarcomas of the gynecologic tract demonstrated that there was evidence of increased overall survival and improved local control after surgery and adjuvant radiation therapy in cases arising from the uterus, vagina, and vulva [4]. For ovarian angiosarcomas or advanced metastatic angiosarcomas, chemotherapy may play a key role in overall survival benefit [9], but its efficacy in primary vaginal angiosarcomas is yet to be determined and warrants further investigation.

## Conclusions

In summary, primary vaginal angiosarcomas with no prior history of radiation are exceedingly rare. The epithelioid variant of this tumor is even less common, with just a handful of cases reported in the literature so far. The rarity of this entity poses a diagnostic challenge as well as contributing to the lack of consensus regarding its optimal management. Currently, wide surgical resection and adjuvant radiotherapy remain the mainstay of treatment. The prognosis remains poor, with a median survival of roughly seven months after diagnosis.
